# A strong and deformable *in-situ* magnesium nanocomposite igniting above 1000 °C

**DOI:** 10.1038/s41598-018-25527-0

**Published:** 2018-05-04

**Authors:** Sravya Tekumalla, Yogesh Nandigam, Nitish Bibhanshu, Shabadi Rajashekara, Chen Yang, Satyam Suwas, Manoj Gupta

**Affiliations:** 1 Department of Mechanical Engineering, National University of Singapore, 9 Engineering Drive 1, Singapore, 117576 Singapore; 2 Department of Metallurgical and Materials Engineering, Indian Institute of Technology Roorkee, Uttarakhand, 247667 India; 3 Department of Materials Engineering, Indian Institute of Science, Bangalore, 560012 India; 4 Laboratory of Physical Metallurgy and Materials Engineering, Unité Matériaux Et Transformations, UMR CNRS 8207, Université Lille – 1 Sciences et Technologies, 59650 Villeneuve d’Ascq, France

## Abstract

Magnesium has been trending of late in automobile, aerospace, defense, sports, electronic and biomedical sectors as it offers an advantage in light-weighting. In aluminum, titanium, and steel dominated aerospace and defense sectors, applications of Mg were banned/restricted until recently due to perceived easy ignition and inability to self-extinguish immediately. Strength is generally inversely related to ductility, weak texture and unrelated to ignition resistance, making it challenging to optimize all four concurrently in a material. We address this challenge by designing a low density (~1.76 g.cm^−3^) *in-situ* Mg nanocomposite. It is a resultant of a sequence of *in-situ* reactions during melt processing and extrusion. The *in-situ* formed Y_2_O_3_ nanoparticles exhibit coherency with matrix and lead to development of large amount of elastic and plastic strain fields around them. These nanoparticles and secondary phases (Mg_2_Ca and Mg_2_Y) are responsible for the nanocomposite’s high tensile strength (~343 MPa). A weak texture mediated tensile ductility of 30% and compressive failure strain of 44% is observed. Further, the ignition temperature increased to 1045 °C (near the boiling point of Mg)  due to the formation of protective surficial oxide layers aided by the presence of insulating Y_2_O_3_ nanoparticles, rendering the nanocomposite outperform other traditional commercial Mg-based materials.

## Introduction

Reduction in weight of aircrafts and rockets in transportation sector accounts for a pronounced increase in the fuel efficiency and is an assertive route to restrict the emissions (emissions from transportation sector constituting up to 14% in 2010 & 26% in 2014)^1^. Replacement of the currently dominant aluminum, titanium alloys and steels will turn out to be a game changer in this field, primarily in view of the coarsening climate change. For decades now, research is being done on developing suitable light weight, high performance magnesium alloys^2,3^. However, we are not fully equipped with magnesium technology to suit the automobile, aviation and defense industries. The factors limiting the implementation of these materials are: (i) a limited toughness under static and dynamic loading due to a strong texture; (ii) high susceptibility to ignition (reason for Mg to be popular in pyro applications); and (iii) poor corrosion resistance.

Amongst these three limiting factors, the corrosive tendencies of magnesium alloys can be curbed by employing the coating technology^4^ and by incorporating the right choice of alloying elements^5–7^. While the corrosive tendencies of magnesium alloys can be controlled to an extent, their high flammability continues to remain a problem. The fire extinguishers available today in commercial aircrafts cannot extinguish the fire caused due to the burning of Mg as Mg fire is highly reactive and intensifies over time^8^. It is commonly known that an increase in corrosion resistance occurs with a concurrent sacrifice of mechanical strength^9^. Although a similar relationship has not yet been certainly established for the ignition characteristics, like corrosion, it is also predominantly controlled by oxidative mechanisms. Besides, there is a dearth of Mg alloys that satisfy the criterion of mechanical suitability (strength and toughness) coupled with non-flammable characteristics; giving rise to a ban on the usage of Mg in aircrafts by Federal Aviation Administration (FAA)^10^. In 2014, the ban was lifted (except for the primary structure^11^) since Mg alloys like Elektron 43 and Elektron 21 satisfied the requirements of FAA^12^. However, since very limited alloys satisfy this criterion, there is a large scope for developing suitable ignition and fire resistant magnesium based materials.

The governing mechanism of ignition of Mg alloys is very different as compared to the aircraft materials like Al alloys (AA7075) and Ti alloys (Ti6Al4V). Al and Ti develop protective surface layers and therefore show no sight of ignition until 2072 °C and 850 °C^13,14^, respectively. In comparison, Mg has a poor Piling Bedworth ratio, forming non-adherent and non-protective surface layers which result in ignition in solid state, when the Mg vapor pressure is sufficient. To address this, several attempts in terms of modifying alloy chemistry and geometrical factors have been made, however, with limited attention paid to composites^15^. Composite materials^16,17^, with their high specific stiffness and low coefficient of thermal expansion (CTE), provide the necessary characteristics to produce lightweight and dimensionally stable structures in aircraft and spacecraft missions. Nanocomposites of Mg base despite having a sound combination of mechanical and thermal stabilities^15,18^, remained largely unexplored in the field of ignition resistant materials and the underlying mechanisms are unclear. By exploiting the thermodynamic feasibility of the reactions, hence forming nanoparticles *in-situ*, novel *in-situ* fabrication of magnesium based nanocomposites is designed. The *in-situ* Mg-1.8Y/1CaO (wt.%) nanocomposite, designed to overcome the limitations of poor strength, ductility, strong texture and ignition tendency, is synthesized using disintegrated melt deposition technique^17^ and thermo-mechanically processed by extrusion at 350 °C. It is to be noted that the microstructure and properties reported henceforth are for the as-extruded materials.

## Results and Discussion

### Holistic outline of properties

**Figure 1:**
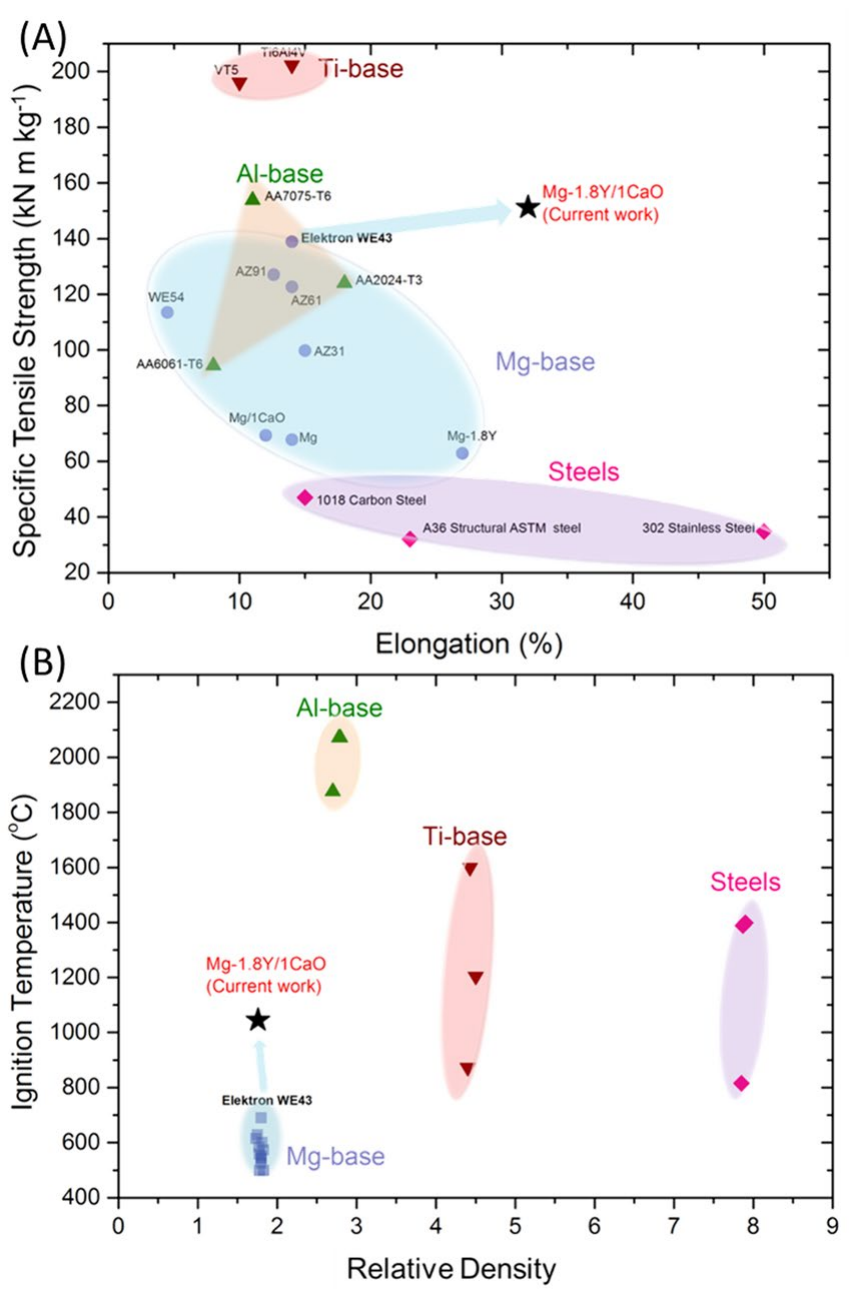
Holistic properties constituting specific strength-ductility-ignition temperature of the Mg-1.8Y/1CaO nanocomposite in comparison to the commercially available alloys. (**A**) Density normalized tensile yield strength versus elongation to failure of nanocomposite in the current work compared with commercially used alloys in aerospace applications; **(B)** An overview of the ignition properties and relative density of the commercial metallic materials. Ignition temperature versus relative density of commercial Mg-base alloys (AZ31, AZ91, Elektron WE43), commercial Al-base alloys (AA2024, AA6061 and AA7075), commercial Ti-base alloys (Ti6Al4V, VT5 alloy) and commercial steels (302 stainless steel, 1018 stainless steel, A36 structural steel).

Figure 1 shows the combination of properties exhibited by the extruded nanocomposite. It exhibits very high specific tensile yield strength (~152 kN.m.kg^−1^), elastic modulus of 45 GPa and a large tensile elongation (~30%) at room temperature, as compared to commercial Mg alloys with specific strength of 60–130 kN.m.kg^−1^. Consequently, the nanocomposite has an extremely high toughness (of 101 MJ/m^3^ ~7 times higher than that of pure Mg)^19^ which is an indicative of its promising bend-before-break ability. In the aerospace and defense sectors, materials are typically subjected to dynamic stresses and to be able to withstand loads without failing is imperative for Mg alloys in order to compete with the contemporary Al and Ti alloys. Under dynamic conditions, conventional Mg alloys usually fail at elongations less than 25%^20^. Before failure, the Mg-1.8Y/1CaO has an excellent deformability under both static and dynamic compression withstanding strains up to 44% and 41% respectively. Figure 1A shows that the Mg-1.8Y/1CaO nanocomposite displays far superior mechanical performance in comparison with the FAA approved Elektron WE43 alloy^21^ and conventionally used Mg, Al, Ti and Fe based commercial alloys^13,14,22–27^, thus validating its unparalleled mechanical suitability.

Simultaneous existence of mechanical sturdiness and non-susceptibility to ignition is an important criterion, yet extremely challenging. Figure 1B, Fig. S6A and Table S1 show an ignition temperature of 1045 °C for Mg-1.8Y/1CaO nanocomposite, which is only marginally lower than the boiling point of magnesium (1091 °C). A holistic outline of the candidate materials^13,14,22–27^ in terms of relative density and ignition temperature is given in Fig. 1B. Since the melting temperature of Mg is 650 °C, it is presumed that Mg auto-ignites at or below 650 °C. However, modification of the chemistry by incorporation of Y and CaO altered the reaction sequence at the surface of the nanocomposite when exposed to high temperatures, which is discussed in the subsequent sections.

### *In-situ* evolution of a multi-component structure

**Figure 2:**
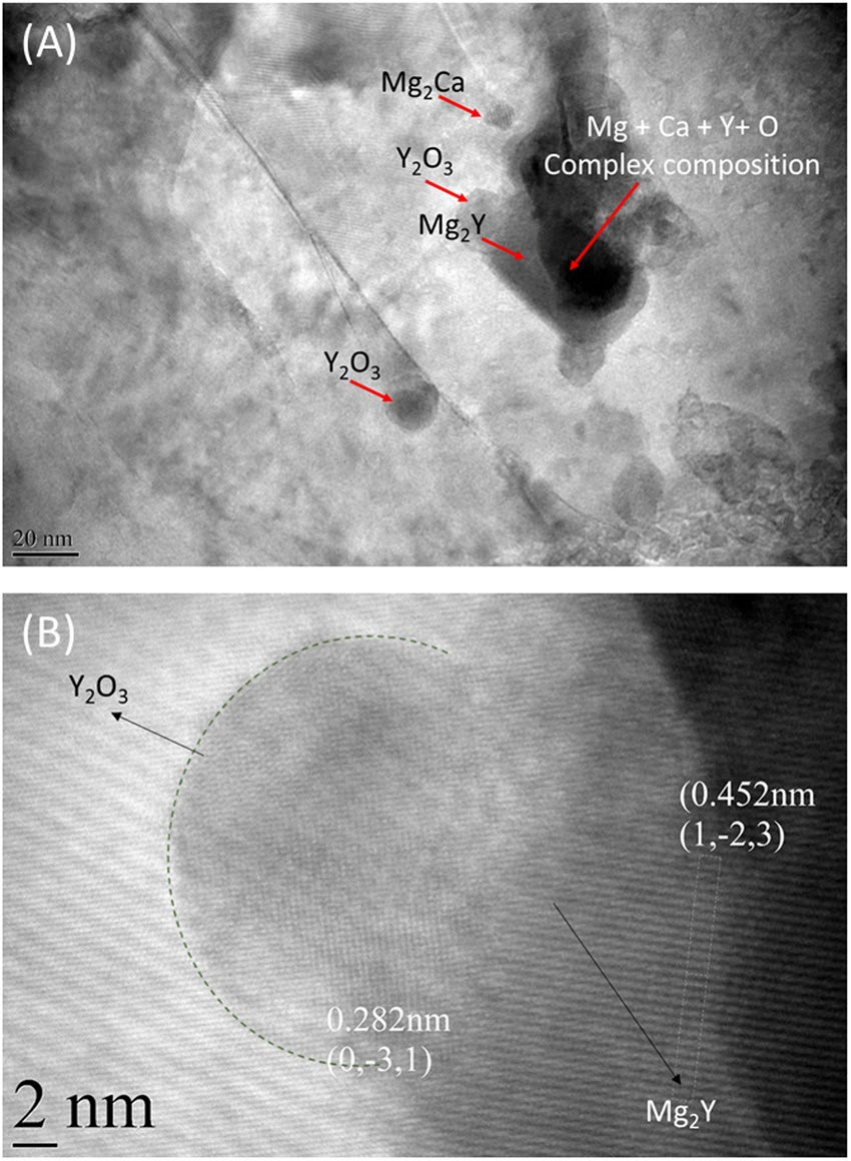
High-resolution transmission electron micrographs of the nanocomposite. (**A**) Distribution of phases and reinforcement in the matrix of the Mg-1.8Y/1CaO nanocomposite; and (**B**) a matrix-reinforcement interface showing the yttria nanoparticle emerging out of Mg_2_Y phase.

We used DSC and TEM to evaluate the microstructure and phases present in the nanocomposite. From the DSC curves (Fig. S3A), it is explicit that the nanocomposite undergoes transformation vis-a-vis both Mg-1.8Y and Mg/1CaO: indicating the presence of Mg-Y phase and Mg_2_Ca phase (transformation at ~407 °C^28^). Through a detailed TEM evaluation (Figs 2A,B, S4A,B), the following are concluded:

(i) undiscernible clusters of Mg + Y + Ca + O (Fig. 2A) with complex compositions - result of a possibly incomplete transformation;
(ii) striking presence of spherical Y_2_O_3_ particles of the order of 15–20 nm length scale, an indication of an *in-situ* reaction sequence at the matrix-reinforcement interface (Fig. 2A and 2B);
(iii) a resultant of an incomplete reaction where the spherical Y_2_O_3_ emerge out of Mg_2_Y phase (submicron − 1 micron size range) (Fig. 2B); and
(iv) a few CaO nanoparticles (40 nm) intact in the matrix (Fig. S4B) while a few reacting with the Mg-1.8Y matrix by dissolving to form barrel shaped Mg_2_Ca of the order of 10–20 nm (Fig. S4B).

These results are consistent with the previous studies on Mg-CaO system where transformation of CaO to Mg-Ca phase occurs^28,29^. However, in the current work, due to the presence of Y in the matrix, formation of brittle MgO can be avoided. Instead, formation of Y_2_O_3_ (nano sized and spherical shaped) occurs as a consequence of higher reactivity of Y leading to *in-situ* reactions between matrix and reinforcement. Similar observations of reactions were observed in a ZnO introduced Mg-1.8Y alloy^30^. These *in-situ* formed stable nano Y_2_O_3_ and Mg_2_Ca ascertain the mechanical and thermal behavior of the nanocomposite, as discussed in the subsequent sections.

**Figure 3:**
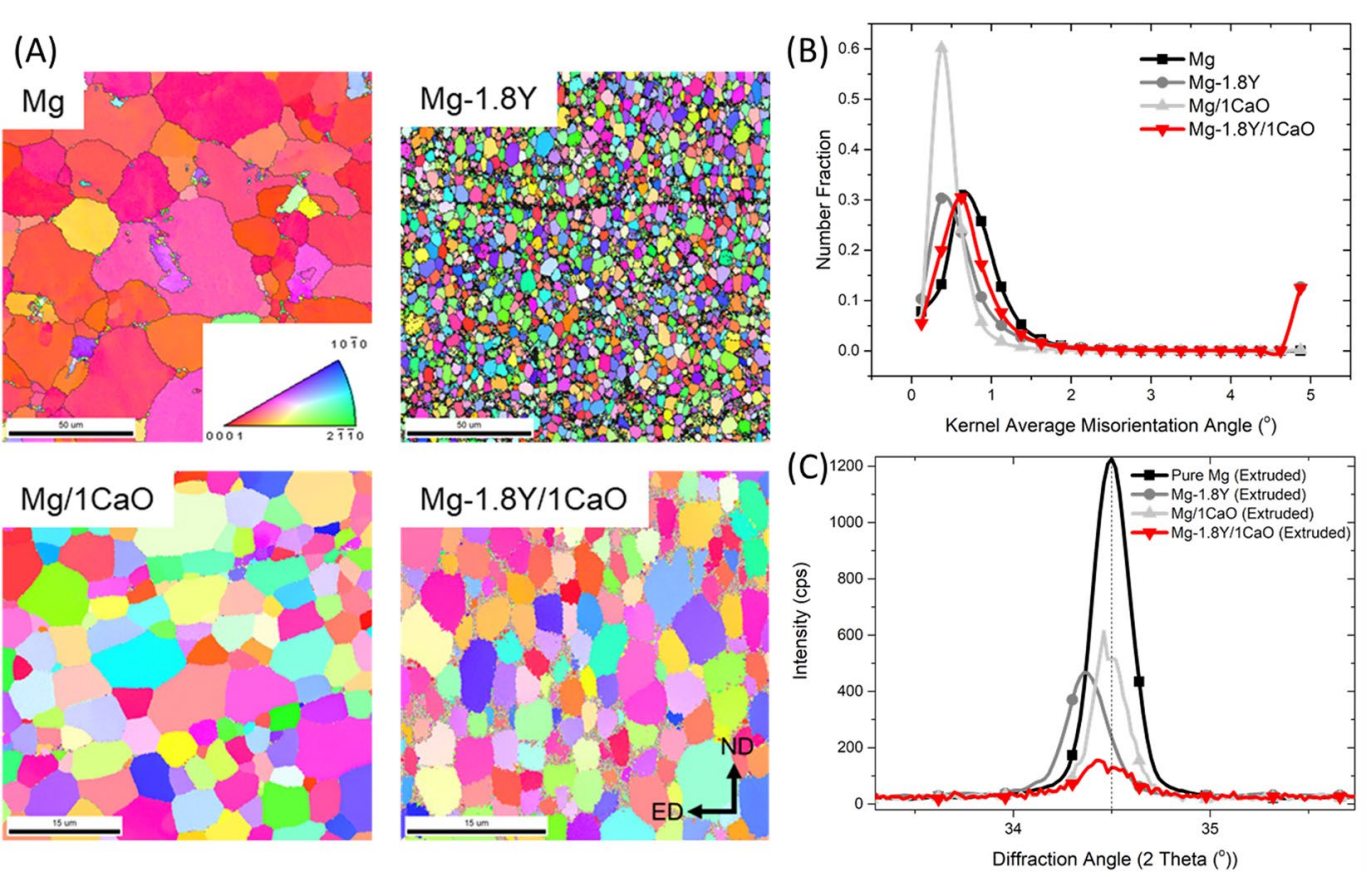
(**A**) Electron back-scattered diffraction patterns of the materials (**B**) Kernel Average Misorientation plots of the materials (**C**) X-ray diffraction results of the nanocomposite in comparison with its monolithic material. It must be noted that the difference in intensities correlated to the texture of the materials and is given in arbitrary units of counts per second.

Other key structural aspects like the final grain size and texture also are distinct in the nanocomposite in comparison to the monolithic materials. It is observed from EBSD Inverse pole figure maps (IPF) (Fig. 3A and Fig. S2) that the final grain size of Mg-1.8Y/1CaO is 6.3 ± 0.9 µm which is marginally higher than the average grain size of Mg-1.8Y (5.4 ± 0.6 µm), but lower than Mg/1CaO (8.3 ± 1.8 µm) and pure Mg (22.7 ± 9.8 µm). Unlike in pure Mg, the grains in Mg-1.8Y/1CaO seemed to be more equi-axed and along the axial direction of the extruded rod. The combined addition of Y and CaO in the matrix led to a decreased grain size as compared to Mg and Mg/1CaO. However, the reason for an increase in the grain size compared to the Mg-1.8Y alloy is thought to be the reaction sequence at the interface of matrix and reinforcement^31^, resulting in less effect of grain refinement.

Further, all the materials are seen to have dynamically recrystallized microstructure. This can be affirmed through the kernel average misorientation (KAM) plots given in Fig. 3B. The greater extent to which the peak shifts towards the origin with an increased number fraction, the more the material is said to be recrystallized^32^. Since, the peaks for all the materials were at a misorientation angle lower than 1°, the materials can be considered to be recrystallized. From the IPF maps (Fig. 3A) and the inverse pole figures (Fig. S3B), it can be seen that Mg-1.8Y, Mg/1CaO and Mg-1.8Y/1CaO have relatively weaker textures as compared to the strong basal fiber in the texture of Mg (Typically, magnesium displays a strong extrusion texture which makes the material hard to deform along certain orientations). It is also ascertained that Mg-1.8Y/1CaO has the weakest texture (see intensities in Fig. S3B) in comparison to the other materials. Further, presence of a weak prismatic fiber is also seen in the nanocomposite, which is attributed to the effect of Y. This attribute results in exhibition of significantly high ductility by the nanocomposite^33^ and renders the material suitable for applications involving extensive deformations.

### Structural genesis of the mechanical properties

*In-situ* evolution of this multicomponent structure in the nanocomposite resulted in excellent properties. In magnesium alloys and nanocomposites, strengthening mechanisms like Hall-Petch strengthening are most commonly the reason to achieve higher yield strengths. The grain size of the nanocomposite is not significantly different from that of the alloy (Fig. S2), however the yield strength is more than double, an indication of dominance of strengthening mechanisms other than the Hall-Petch strengthening. Further, it is established that weaker textures in magnesium alloys (as obtained in this current study for the nanocomposite) result in lower yield strengths. However, the yield strength of the nanocomposite is very high. This is analysed to be due to the presence of the *in-situ* formed secondary phases as well as nanoparticles.

Y_2_O_3_ in the order of 15–20 nm length scale along the grain boundaries (Fig. S4A) interacts with grain boundaries, by acting as pinning sites and retards the dislocation motion. Further, there is a need to identify the internal strains induced in the nanocomposite due to the nanoparticles, to ascertain its high strength. The elastic component of the strain was analysed using XRD, while the plastic component was identified using KAM, which is an indirect measure of the geometrically necessary dislocations in the material. Addition of Y to Mg shifts the XRD peak at 34.5° to left (Fig. 3C), however with Y and CaO addition, the peak shifts to the right of the alloy and nearly coincides with that of Mg. This reveals that the nanocomposite has a higher elastic micro-strain as compared to that of the alloy. Further, from the KAM plot, it is also seen that Mg-1.8Y alloy and Mg/1CaO exhibit the least plastic micro-strain (shift in the peak to the left), while Mg and Mg-1.8Y/1CaO exhibit a higher micro-strain, which is mainly due to the recrystallization response of the materials as discussed in the previous section. The micro-strain results from KAM and the XRD data are an indicative of the presence of substantial elastic and plastic strains in the matrix in the nanocomposite. This is a result of the *in-situ* formation of nanoparticles, where the nanoparticles originate out of the phase through an exothermic reaction leaving a coherent interface as shown in Fig. 2B. In Y_2_O_3_ reinforced Mg nanocomposite reported by Goh *et al*.^34^, an incoherent interface was observed, while this nanocomposite, with *in-situ* formed Y_2_O_3_, had a coherent interface with the matrix. This results in the substantial presence of strain fields around the nanoparticles, hence, an increase in the resistance to dislocation motion resulting in high yield strength. Thus, the *in-situ* formation of the nanoparticles contributed to an overall enhancement in its toughness and mechanical functioning.

### Structural genesis of the ignition properties

**Figure 4:**
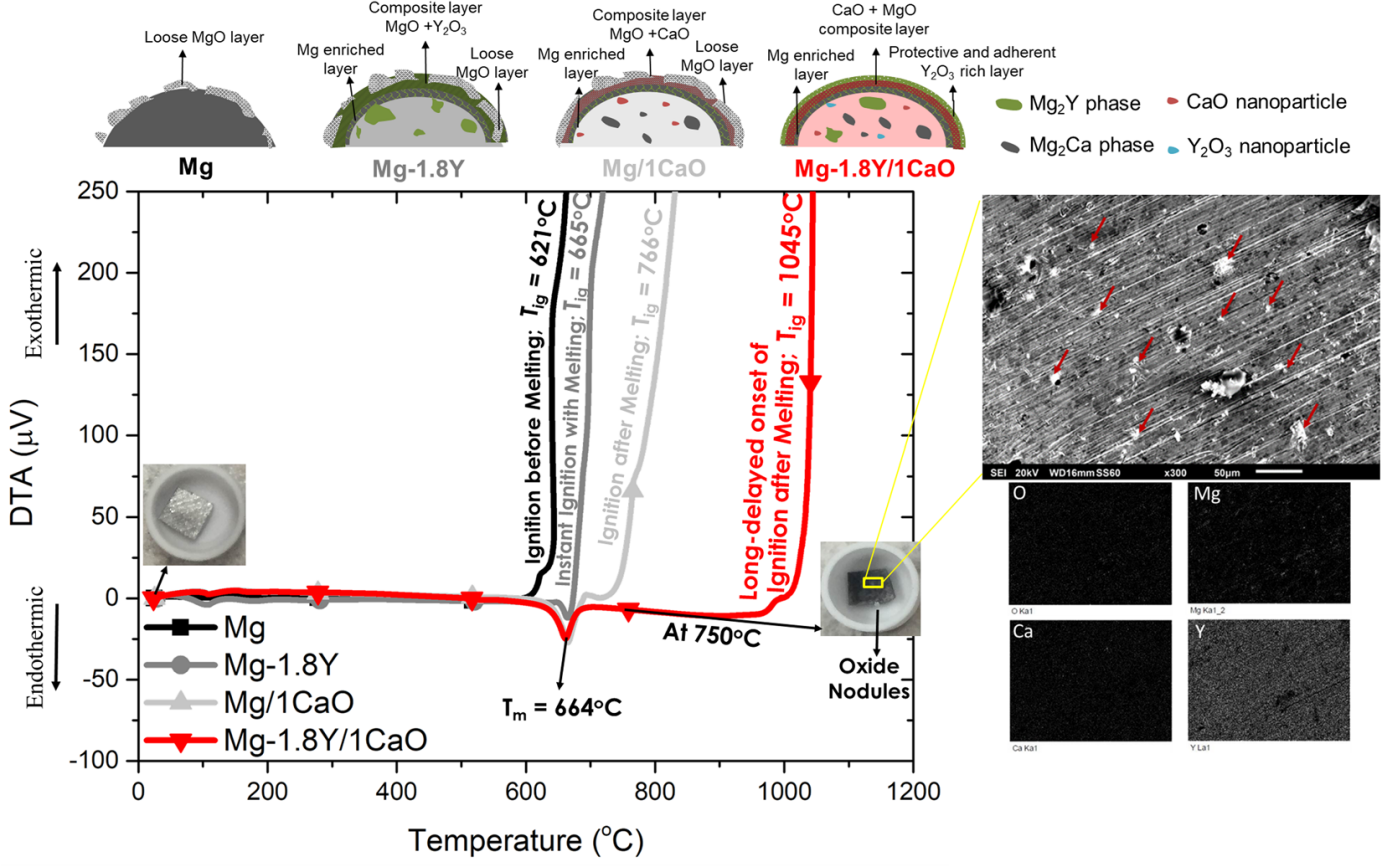
Schematic of DTA vs Temperature of all the materials. Endothermic peak indicates the melting temperature of the materials. Mg curve represents ignition before melting due to the presence of loose MgO layer; Mg-1.8Y alloy shows instant ignition with melting due to the presence of Y_2_O_3_ rich oxide scale; Mg/1CaO ignites after melting due to a more protective CaO + MgO scale and Mg-1.8Y/1CaO nanocomposite with the presence of multiple secondary phases and nanoparticles (not drawn to scale) developed *in-situ* develops a completely protective oxide layer that exhibits long-delayed onset of ignition. An inset of the sample heated until 750 °C in TGA and cooled down to room temperature shows an unignited Mg-1.8Y/1CaO. X ray mapping of the image indicates the dominating continuous presence of Y_2_O_3_ with a distributed presence of CaO and MgO layer on the surface indicating a Y_2_O_3_ rich surface.

The dominant mechanisms determining the ignition temperatures of magnesium based materials are complex and are not very well known, especially of the nanocomposites. The term ‘ignition’ is often misconstrued with terms like oxidation, melting, etc. The authors would like to clarify that ignition is the tendency of a material to burn instantly without being able to self-extinguish immediately. Materials like Al, despite having melting temperatures as low as 660 °C, have ignition temperatures greater than 2000 °C, i.e. Al does not burn instantly after melting due to formation of protective oxide layers. However, the same is not the case with traditional magnesium materials as they auto-ignite at temperatures lower than the melting temperatures (see Table S1), which is the reason for their non-applicability. For instance, pure Mg, as shown in Fig. 4, when subjected to slow heating in air, ignites completely at 621 °C even before melting (no melting/endothermic peak in curve corresponding to Mg). This is due to the high susceptibility of magnesium to oxidize at higher temperatures. The addition of yttrium lead to increased melting and ignition temperature of magnesium, leading to instant ignition with melting of the alloy at 665 °C. With addition of only CaO to Mg, the material ignited slightly after melting at 766 °C. In Mg-1.8Y/1CaO nanocomposite, a long-delayed onset of ignition occurred at 1045 °C despite the fact that the material had the same melting temperature as that of Mg-1.8Y and Mg/1CaO i.e. 664 °C. This is interesting, considering the fact that some Mg materials burn much before melting. This is a breakthrough in research for ignition resistant materials as this mechanism is akin to that of aluminum based materials. However, the two cannot be compared figuratively, as the boiling temperatures of Mg (1091 °C) and Al (2470 °C) are wide apart, thus leaving a huge disparity in the maximum ignition temperatures that can be achieved. This work achieved in getting very close to the boiling temperature of Mg by reaching ignition temperature of 1045 °C.

The detailed mechanism is discussed (see Fig. 4) as follows: when the bulk Mg-1.8Y/1CaO nanocomposite (with Mg_2_Y, Mg_2_Ca, CaO and Y_2_O_3_ dispersed in α-Mg matrix) is exposed to increasingly high temperatures, oxidation kinetics plays a dominant role. The preliminary oxide layer formation depends on the diffusion of the ions based on the diffusion coefficient data^35^. Mg^2+^ ions (smaller) diffuse faster than the Y^3+^ and Ca^2+^ ions (larger) and react with O^2−^ at the gas/metal interface leading to the formation of a thin outermost layer of MgO (Fig. 4). This causes an Mg-depleted zone underneath giving rise to an yttrium enriched layer (Fig. 4). Formation of yttrium rich oxide beneath MgO layer at the metal/oxide interface occurs until 390 °C (from the DSC results in Fig. S3A, the complete dissolution of phase occurs at 390 °C). As temperature increases, the dissolution of CaO to form Mg_2_Ca occurs at 407 °C, leaving scope for the diffusion of Ca^2+^ ions as well through the layer at metal/oxide interface. Beneath these layers, another layer is feasible to form due to the faster diffusion of Ca^2+^ as compared to Y^3+^ on the nanocomposite surface with a complex composition of MgO, CaO and Y_2_O_3_ (Fig. 4). Thus formed dense composite oxide scale slows down the outward diffusion of Mg^2+^. With increasing temperatures, the MgO layer loses its protective nature and breaks away leaving behind the Y_2_O_3_ rich layer. This stable layer prevents the exposure of Mg to oxygen resulting in slower increase in surface temperature.

Based on the SEM results (Fig. 4) of the surface analysis of sample heated until 750 °C, Y_2_O_3_ rich layer is stable until a much higher temperature as compared to MgO and at 750 °C. In addition, the surface of the nanocomposite indicated presence of localized oxide nodules of MgO (Fig. 4), some visible to the eye. When the temperature increases further, the surface layer becomes loose leading to further exposure of these nodules to the air. Due to the exothermic nature of the oxidation process, the high localized heat energy released causes evaporation of Mg, when these Mg vapors come in contact with air, ignition of the nanocomposite occurs. Alongside the surface oxide layers of Y_2_O_3_, the *in-situ* evolved thermally stable Y_2_O_3_ nanoparticles (insulators) delay the onset of ignition by not allowing the heat transfer to take place in the matrix across the nanoparticles. Though the modification of alloy chemistry cannot prevent the complete ignition of the material, it delays the onset of ignition until close to its boiling temperature. Further elaborate discussion in comparison to the monolithic alloys is given in the Supplementary material.

### Multitudinous functions of yttrium

Yttrium plays a multitudinous role in the excellent functioning of the nanocomposite. Firstly, it assists in the reduction of CaO into the matrix. From the Ellingham diagram (free energy versus temperature plots), it can be seen (Fig. S8) that the line of 2Y + 3 O → Y_2_O_3_ is under the Ca + O → CaO line at 750 °C (melting temperature) (however, the two lines are close at that temperature). This could possibly be the reason for partial reduction of CaO as the kinetics also plays a crucial role. Very few other elements like Tm (Thulium) satisfy this criterion (Fig. S8). This helps in the dissolution of CaO and preferential oxidation of Y to form Y_2_O_3_. These Y_2_O_3_ particles enhance the ignition temperature and strength in the nanocomposite by providing a non-conductive interface for heat transfer as well as by being hard obstacles for dislocation motion. Secondly, Y promotes activity of non-basal slip as shown in Figs 3A and S3B and established previously^36^. This results in the excellent deformability of Mg under both static and dynamic conditions^37^. Thirdly, it aids in the modification of the surficial oxide layer. The formation of a dense compact oxide layer of Y_2_O_3_ (mainly), CaO and MgO is possible due to the addition of Y and its synergistic effect with Ca to form a stable oxide layer^38^. Each or two of the afore mentioned roles may be displayed by other alloying elements as well, however, the collective role of all the three functions can be displayed by only Y, thus making the designed composition, for the nanocomposite, unique.

### Competition with commercial structural materials

**Figure 5:**
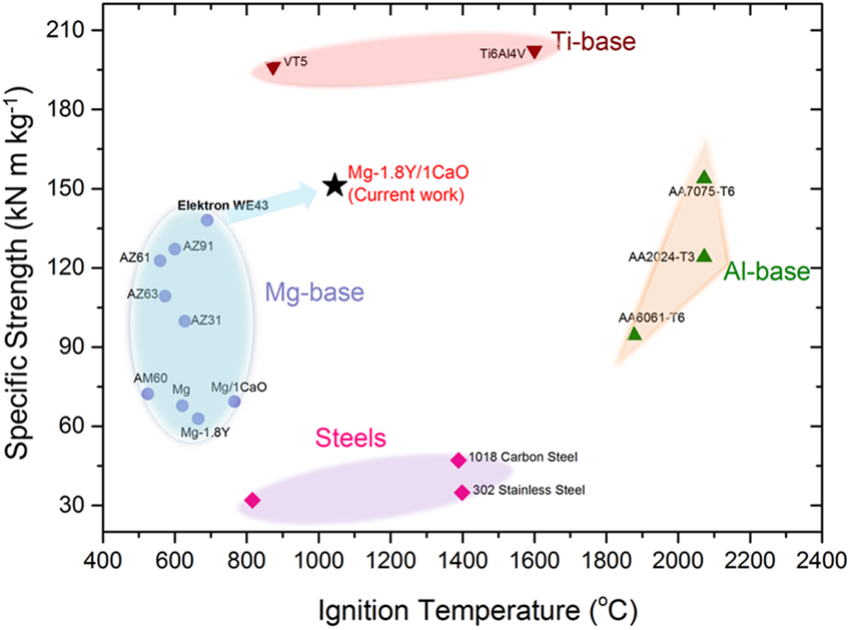
Density normalized tensile strength versus ignition temperature of Mg-1.8Y/1CaO in comparison with the commercial Mg-base alloys, Al-base alloys, Ti-base alloys and steels.

The nanocomposite with such superior properties is fabricated by a fairly simple process of casting and extrusion, similar to that of the Al and Ti alloys. The novel *in-situ* reactions taking place during the processing aid in uniform and homogeneous dispersion of nanoparticles in magnesium matrix, which otherwise is not possible in traditional Mg nanocomposites fabricated using conventional routes. Thus, the *in-situ* nanocomposites of Mg render the processing industrially viable using standard routes of processing. Plots of ignition temperature versus specific strengths in various commercial alloys are shown in Fig. 5. The present Mg-1.8Y/1CaO nanocomposite possesses a relative density of 1.76 and its specific strength is much higher than that of steels and most Mg alloys and Al alloys, while its deformability is at par with/exceeding Al, Ti base alloys and steels. Its ignition temperature (1045 °C) is only slightly lower than the boiling temperature and is much higher than FAA approved Elektron WE43 alloy (690 °C) and comparable to that of Ti base alloys and steels and lower than Al base alloys. It is almost practically impossible to raise the ignition temperature of Mg alloys/nanocomposites beyond the boiling temperature. It may be noted that Mg suffers from a poor boiling temperature of 1091 °C, while Al and Ti boil at 2470 °C and 3287 °C, hence reaching high ignition temperatures like that of Al is impracticable. This lightweight, high strength, ductile, 100% recyclable and ignition resistant nanocomposite has a great potential in defense and aerospace applications, and can be extended to others including automotive, electronic, sporting and biomedical applications. Further, employing this particular material combination can also help prevent the usage of environmentally unfriendly cover gases during material fabrication as the nanocomposite develops a very stable dense protective layer that can inhibit the initiation of fire even during melting^39^.

## Conclusions

In short, an *in-situ* Mg-1.8Y/1CaO nanocomposite with superior mechanical (tensile strength ~343 MPa, ductility ~30%) and ignition temperature (1045 °C) has been reported for the first time. A thermodynamically reactive matrix with Y as an alloying element to Mg is chosen such that it aids in the *in-situ* reaction as well as imparts ductility to the matrix by weakening texture. The CaO reinforcement reacts with the Mg-1.8Y matrix at processing temperatures to form *in-situ* Mg_2_Ca and *in-situ* Y_2_O_3_ nanoparticles with a coherent interface with the matrix. Despite a weak texture, due to the presence of substantial elastic and plastic strain fields around the nanoparticles, the nanocomposite exhibited high strengths. Further, the presence of thermally insulating Y_2_O_3_ nanoparticles and formation of surficial complex oxide layers delayed the onset of ignition until 1045 °C.

This finding is a breakthrough for the budding magnesium composite technology and broaches a potential for the progress in research towards magnesium based nanocomposites in the interest of light-weighting and suggests industrially scalable new *in-situ* synthesis of nanocomposites.

## Methods

### Primary processing

Mg-1.8Y/1CaO (wt.%) nanocomposite was synthesized by melting and casting commercially pure Mg turnings (99.9% purity; supplied by Acros Organics, USA), Mg-30% (wt.%) Y master alloy (99% purity; supplied by Sunrelier Metal Co. Limited, China) and CaO nanoparticles (Nanoshel LLC, USA; 40 nm average size). The synthesis technique employed is the DMD (Disintegrated Melt Deposition) method^17^. This method involves adding the raw material (i.e. Mg, Mg-30Y and CaO) in alternate layers to form a sandwich pattern in a graphite crucible and heating it in an electrical resistance furnace to 750 °C in a protective inert argon gas atmosphere. This method employs a combination of vortex stirring of melt at 450 rpm for 5 minutes. The stirrer used was a mild steel impeller with twin blade (pitch 45°) coated with Zirtex 25 (86% ZrO_2_, 8.8% Y_2_O_3_, 3.6% SiO_2_, 1.2% K_2_O and Na_2_O, with 0.3% trace inorganic) in order to avoid contamination of molten metal with iron. The melt, released through an orifice of 10 mm diameter, located at the crucible’s base was disintegrated by two argon gas jets that were oriented normal to the melt stream to obtain near equi-axed grain structure. The disintegrated melt was then deposited on the substrate forming an ingot of 40 mm diameter.

### Secondary processing

Billets (length: 45 mm and diameter: 36 mm) of Mg-1.8Y/1CaO nanocomposite obtained from DMD technique were homogenized at 400 °C for 1 hour and extruded at 350 °C to obtain rods of 8 mm diameter, hence maintaining an extrusion ratio of 20.25:1. Samples were taken from these rods for further analysis.

### Grain size measurement

Grain size of each material was obtained using the linear intercept method following the ASTM E112–13 standard using images from an optical microscope (Olympus) on polished and etched samples (etchant: 60 ml ethylene glycol, 20 ml acetic acid, 1 ml nitric acid and 20 ml distilled water).

### X Ray Diffraction

An automated Shimadzu LAB-XRD-6000 (Cu Kα; λ = 1.54 A°) spectrometer with a scan speed of 2°/min was used for X-ray diffraction analysis of samples. Macro textures were measured using a Bruker D8-Discover texture goniometer using Co-Kα radiation. During X-ray texture measurements all the samples were irradiated perpendicular to the extrusion direction along the radial plane. Six pole figures viz., (1 0 1 0), (0 0 0 2), (1 $\bar{0}$ 1 1), (1 $\bar{0}$ 1 2), (1 $\bar{1}$ 2 0) and (1 $\bar{1}$ 2 $\bar{2}$) were measured and orientation deformation functions (ODFs) were calculated using ADC algorithm using a Labotex Software. Inverse pole figures were calculated from the ODFs.

### Phase/reinforcement determination

Phase analysis was performed using scanning electron microscopes (JEOL JSM-6010 and Hitachi FESEM-S4300) coupled with energy dispersive spectrometric analysis (EDS) and a 200 kV Tecnai F20 Transmission Electron Microscope (TEM) on polished and etched samples. Slices of 200 µm were taken from each material, thinned mechanically to 50 µm, made electron transparent in a Gatan polisher and a Precision Ion Polishing System (PIPS) with voltage of 3.5 keV and a tilt angle of 7°.

### Micro-texture

Micro-texture of the extruded rods was obtained using the electron back-scatter diffraction (EBSD) technique in a Field Emission Gun Scanning Electron Microscope (FEG-SEM) by FEI Company. The samples were cloth polished with diamond paste (0.05 microns) to obtain a mirror finish, with kerosene acting as a lubricant. Further, mechanical electro-polishing was carried out (using an electrolyte containing 3:5 solution of H_3_PO_4_ in ethanol and pure aluminum cathode at 3 V for 30 s and 1.5 V for 3 min at ~0 °C) in the final polishing step. The EBSD scans were recorded on the radial plane of the samples parallel to the extrusion direction (ED). Kernel average misorientation (KAM) of each EBSD spot with all of its neighbouring spots was calculated with the provision that misorientations exceeding 5° were excluded from the average calculation. The misorientation between a grain at the centre of the kernel and all points at the perimeter of the kernel were measured. The local misorientation value assigned to the centre point was the average of these misorientations, which could be obtained from the EBSD scans. Maps, constructed using this method, are helpful in visualizing the distribution of local misorientation within a grain.

### Phase transformation determination

DSC reaction studies using Shimadzu DSC - 60 instrument was carried out at a heating rate of 5 °C/min from room temperature to 600 °C in flowing argon atmosphere to determine the phase transformations in the alloy and nanocomposites. The deflection in the curves (upward peak or downward peak) indicates the occurrence of reactions due to phase transformations.

### Ignition testing

The ignition temperatures were determined using a simultaneous Thermo Gravimetric Analyzer (TGA)- Differential Thermal Analyzer (DTA). Samples of dimensions 2 × 2 × 1 mm^3^ were heated from 30 °C to 750 °C and 30 °C to 1200 °C with a heating rate of 30 °C/min each in purified air with a flow rate of 50 ml/min. The point at which a rapid increase in the weight of the sample is triggered (as a result of sharp oxidation upon ignition) is considered as the ignition temperature^15^. After the sample was burnt out, the temperature rate was restored to the initial set-value. The crucible was removed immediately after the test, cooled sufficiently in order to avoid contamination of TGA and overflow of the ignited powder from the sample. For each composition, 3 tests were performed to ensure consistency in the results.

### Elastic Modulus Determination

The elastic modulus of the nanocomposite was measured using the resonance frequency damping analyzer (RFDA) equipment from IMCE, Belgium. In this method, the material is manually impulse excited at room temperature. The RFDA basic system measures the resonant frequencies and internal friction or damping of samples and calculates the Young’s modulus according to the ASTM E1876–15 standard. The vibration signal of the material (7 mm diameter, 40 mm length) is recorded and the elastic properties are calculated by the dedicated RFDA basic software.

### Tensile testing

A fully automated servo-hydraulic Model MTS 810 mechanical testing machine was used to determine the tensile behavior of the developed Mg alloys, conforming to ASTM test method E8/E8M-13a. Specimen with a diameter of 5 mm and gauge length of 25 mm were used for the tensile tests and were tested at a strain rate of 1.6 × 10^−4^ s^−1^. A clip-on type extensometer of Instron 2630e100 series was used to determine the displacement/strain. Five tests were performed on each sample to ensure consistency and the representative result is taken in this work.

### Quasi-static compressive testing

Quasi-static compression tests were performed on a fully automated servo-hydraulic mechanical testing machine, Model-MTS 810 conforming to ASTM test method E9-09. Specimen of 7 mm diameter and 7 mm length were tested at a strain rate of 1.6 × 10^−4^ s^−1^. For each composition, 5 tests were performed to ensure consistency in the results. Five tests were performed on each sample to ensure consistency and the representative result is taken in this work.

### Dynamic compressive testing

High-strain-rate compression tests were performed using a Split Hopkinson Pressure Bar (SHPB) at ambient temperature (298 K). In the SHPB setup used in this study, the specimen is sandwiched between the incident bar and the transmission steel bar. The contact end of the specimen/pressure bar was covered with a silicone grease to minimize friction. A high-pressure chamber was used to launch a striker bar, which impacts one end of the incident bar and generates an elastic wave pulse. Strain gauges were bonded on the surface of the incident and transmission bars to detect the propagation of stress waves. Because the stress equilibrium in the specimen was confirmed by the measured stress waves, one wave analysis was conducted in this study. Strain rate $\dot{\varepsilon }$(t), stress σ(t), and strain ɛ(t) were calculated using the following equations^40^.

$$\dot{\varepsilon }(t)=-\frac{2{C}_{B}{\varepsilon }_{B}(t)}{{L}_{0}\,};\varepsilon (t)=-\frac{2{C}_{B}}{{L}_{0}\,}\,{\int }_{0}^{t}{\varepsilon }_{R}(t)dt;\sigma (t)=\frac{{E}_{B}{A}_{B}{\varepsilon }_{T}(t)}{{A}_{0}\,}$$

where C_B_, E_B_ and A_B_ are the elastic wave speed, elastic modulus and cross-sectional area of the bars, respectively; L_0_ and A_0_ are the length and cross-sectional area of the specimen; ε_R_ is the reflected strain, ε_T_ is the transmitted strain, and $\dot{\varepsilon }$ is the strain rate. The average strain rate was measured to be 1.4 × 10^3^ s^−1^ for a specimen of 5.0 mm height. For each composition, 5 tests were performed to ensure consistency in the results.

### Data Availability Statement

All data generated or analysed during this study are included in this published article (and the Supplementary Information files).

## Electronic supplementary material

Supplementary File
