# Supplementary material for: A strong and deformable in-situ magnesium nanocomposite igniting above 1000 °C
Source: Sci Rep. 2018 May 4;8:7038. doi: 10.1038/s41598-018-25527-0 (PMC5935745; doi:10.1038/s41598-018-25527-0)
Supplement: Supplementary file 1 — Supplementary File [file 41598_2018_25527_MOESM1_ESM.pdf]

Supplementary Materials for

A strong and deformable in-situ magnesium nanocomposite igniting above 1000°C

Authors:

Sravya Tekumalla[1*], Yogesh Nandigam[2], Nitish Bibhanshu[3], Shabadi Rajashekara[4], Chen Yang[1], Satyam Suwas[3] and Manoj Gupta[1]*

Affiliations:

[1]Department of Mechanical Engineering, National University of Singapore, 9 Engineering Drive 1, Singapore 117576.

[2]Department of Metallurgical and Materials Engineering, Indian Institute of Technology Roorkee, Uttarakhand, India, 247667.

[3]Department of Materials Engineering, Indian Institute of Science, Bangalore, India, 560012.

[4]Laboratory of Physical Metallurgy and Materials Engineering, Unité Matériaux Et Transformations, UMR CNRS 8207, Université Lille – 1 Sciences et Technologies, 59650-Villeneuve d'Ascq.

*Correspondence to Manoj Gupta: mpegm@nus.edu.sg

This file includes:

Supplementary Text
Figs. S1 to S8
Table S1
Supplementary Reference List

Supplementary Text

Microstructural evolution

We used three different modes of analyses to understand the phases present in the nanocomposite: (i) The DSC in Fig. S3A shows the presence of peaks indicating the occurrence of phases in Mg-1.8Y/1CaO. For the sake of comparison, Mg/1CaO and Mg-1.8Y alloy were also subjected to DSC to see the phases present. In Mg-1.8Y alloy, a peak was seen at ~ 390°C which shows that it belongs to a Mg-Y phase, as indicated previously as well [1]. In Mg/1CaO, a peak was seen at ~ 403°C which is a definite identity for the Mg_2Ca phase that is formed during the melt processing due to the dissolution of CaO nanoparticles in the matrix. It is consistent with the results of a previous study where dissolution of CaO was observed and the temperature of melting of the Mg_2Ca phase was given as 407°C [2]. A small deviation in the temperature is mostly due to the thermal hysteresis. In comparison to both the monolithic counterparts, the peak in Mg-1.8Y/1CaO was observed to be at ~ 390°C which indicates the strong presence of Mg-Y phase in the nanocomposite. A very small peak of Mg_2Ca phase is observed at 407°C. Since the ΔH value is very low, it is assumed that the volume fraction of Mg_2Ca is quite low; (ii) The XRD results in Fig. S3B studied on the cross section of the extruded sample (perpendicular to the extrusion direction) reveals the presence of phases in Mg-1.8Y/1CaO and results show that along with the peaks of α-Mg matrix, there is one very low intensity peak of Mg_2Ca phase, again revealing the extremely low volume fraction of the phase. The peaks of Mg-Y phase were not observed which could be due to the following (a) high solubility of Y in Mg (b) presence of the phase along the basal planes which makes it hard for diffraction of the phases to occur (c) low volume % of phase in the alloy (Cullity [3] has reported that it is difficult for the filtered X-ray to detect the phase when the volume percentage of the phase present in the alloys/nanocomposites is less than 2%); (iii) TEM images in Fig. 2B, 2C and S4 show the presence of Mg_2Ca, Mg_2Y, Y_2O_3, CaO in the magnesium matrix. During the primary processing, CaO and Mg-30Y were added to Mg turnings. However, in the final samples obtained after extrusion, a range of phases and particles are observed. This is an indication of the possibility of a reaction sequence during melt processing as well as during hot extrusion that led to the observed phase patterns.

Ignition Characteristics

Pure Mg can auto-ignite in solid state due to the rapid increase in localized heat that causes melting and evaporation of the metal locally. When the Mg vapor is in contact with air at the gas/metal interface, the metal ignites. The mechanism, in-detail, is indicated in Fig. S6A. However, with the modification of the chemistry of the material i.e. alloying/other additions, the mechanism changes and is studied commonly but still needs further analysis [4]. Addition of a thermally stable alloying element can aid the magnesium matrix in formation of a more stable oxide layer that can delay the onset of ignition. Pilling-Bedworth ratio (PBR), solid solubility of the alloying element as well as the Gibbs free energy of formation are important criterion for the selection of alloying element. Yttrium, satisfying these requirements, is thus chosen. With the single addition of Y to Mg, a composite oxide layer (Y_2O_3+MgO) indicated in Fig. S7D forms. However, due to the lack of a third layer and due to the instability of this composite layer, Y can enhance effectively the ignition temperature until 665°C only. In order to benefit from an additional layer, the reinforcement of nano CaO is added. Nano CaO is chosen because of the following reasons (i) the dissolution of CaO is reported previously, hence providing an easy route for the formation of Ca^{2+} ions that can diffuse outwards; (ii) nano sized reinforcement leads

to a higher spread in the presence of Ca in the matrix rather than a more concentrated presence. This was experimentally proved to be verified as can be seen in the results of the X ray mapping (Fig. 4) which is discussed subsequently. The Y and CaO added Mg-1.8Y/1CaO nanocomposite, when heated to and held at a temperature of 750^oC, did not indicate any occurrence of ignition, surpassing the ignition temperature of Elektron WE43 alloy. When another sample of same composition was subjected to identical heating conditions and heated to a temperature of 1200^oC, the sample indicated a rapid rise in temperature at 1045^oC, which is the ignition temperature of the Mg-1.8Y/1CaO nanocomposite. The surface analysis of unignited sample (heated until 750^oC) was done under SEM (Fig 4), and X-ray mapping detected: (i) a discernibly uniform Y_2O_3 layer was present on the surface (ii) non-uniform distribution of CaO + MgO layer and (iii) a few nodules of MgO (shown using red arrows in Fig. 4). These nodules (protrusions of MgO) are a result of diffusion of oxygen through the Y_2O_3 layer into the sample leading to the oxidation of sub-surficial Mg that forms nodules of MgO. The alloying additions helped in the regeneration of the oxide film until exhausted. This oxide film prevented the outward diffusion of the magnesium ions. With further increase in temperature, these nodules begin to grow on the surface leading to a rapid oxidation and release of higher amounts of heat. These nodules have cauliflower morphology when they grow and are the first regions where the flame occurs due to the easy transfer of Mg vapor through the loose structures, resulting in ignition of the sample at 1045^oC. The detailed mechanism is discussed in the Main text. The powder obtained after ignition of the nanocomposite was subject to XRD analysis. The results are indicated in Fig. S6B. Note that the MgO peak has the highest intensity which is due to the ignition of the bulk sample that forms a powder. The mechanism in monolithic pure Mg, Mg-1.8Y alloy and Mg/1CaO and Mg-1.8Y/1CaO materials are given in Fig S7. These figures show the difference in their mechanisms compared to that of the nanocomposite.

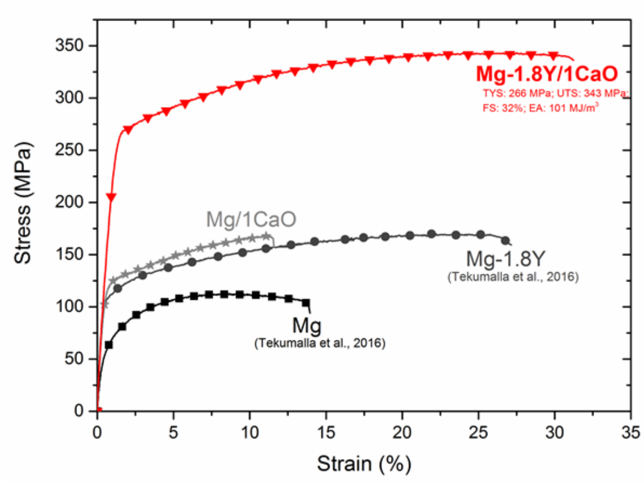

(A)

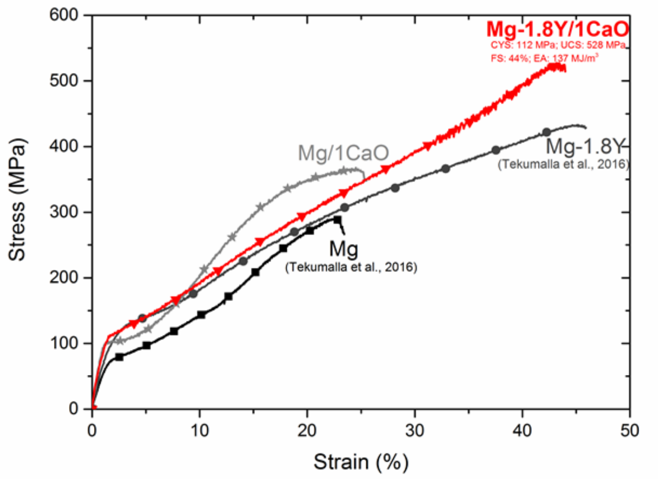

(B)

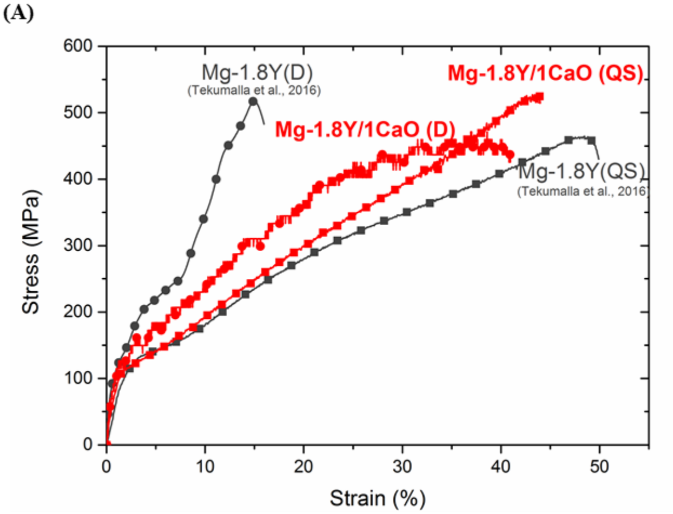

(C)

Fig. S1. (A) Tensile, **(B)** quasi-static compressive and **(C)** dynamic compressive curves of the nanocomposite in comparison to its monolithic alloys. It is to be noted that the dynamic curves are represented with (D) and quasi-static curves are represented with (QS) in Fig. S1C.

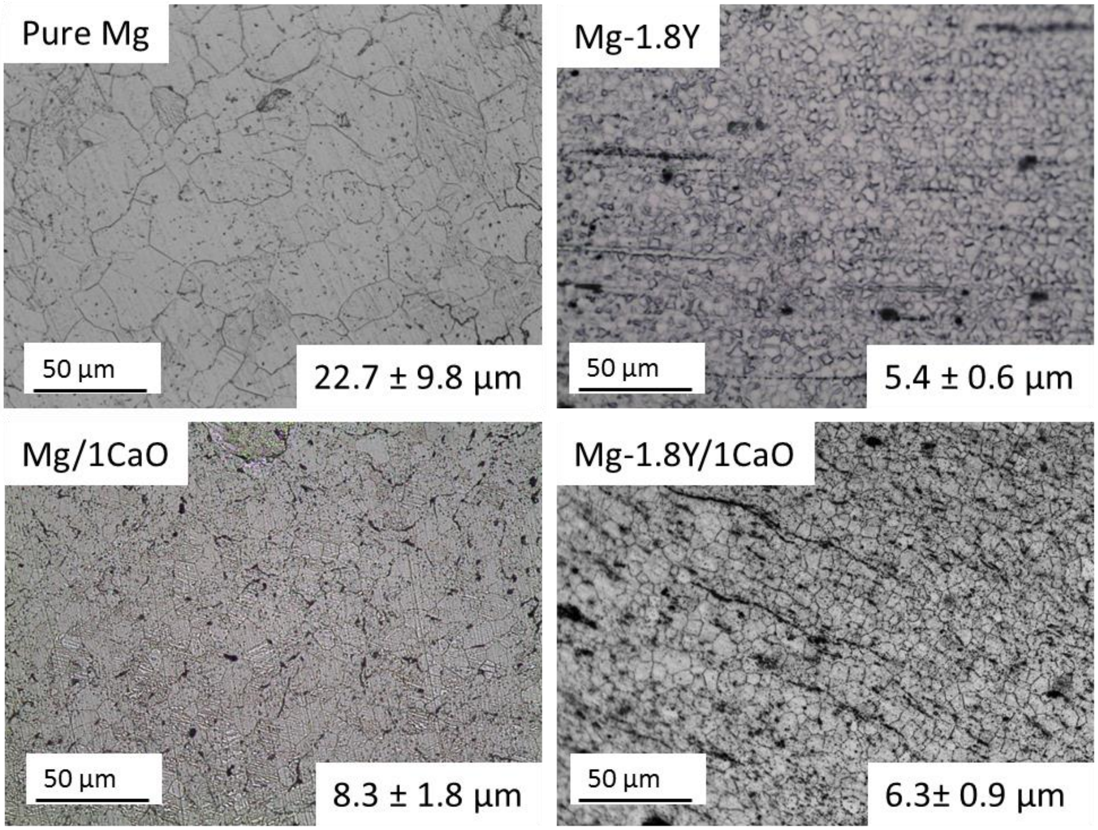

Fig. S2. Optical micrographs indicating the grain sizes of each of the Mg based alloy and nanocomposite as indicated in the insets of the images.

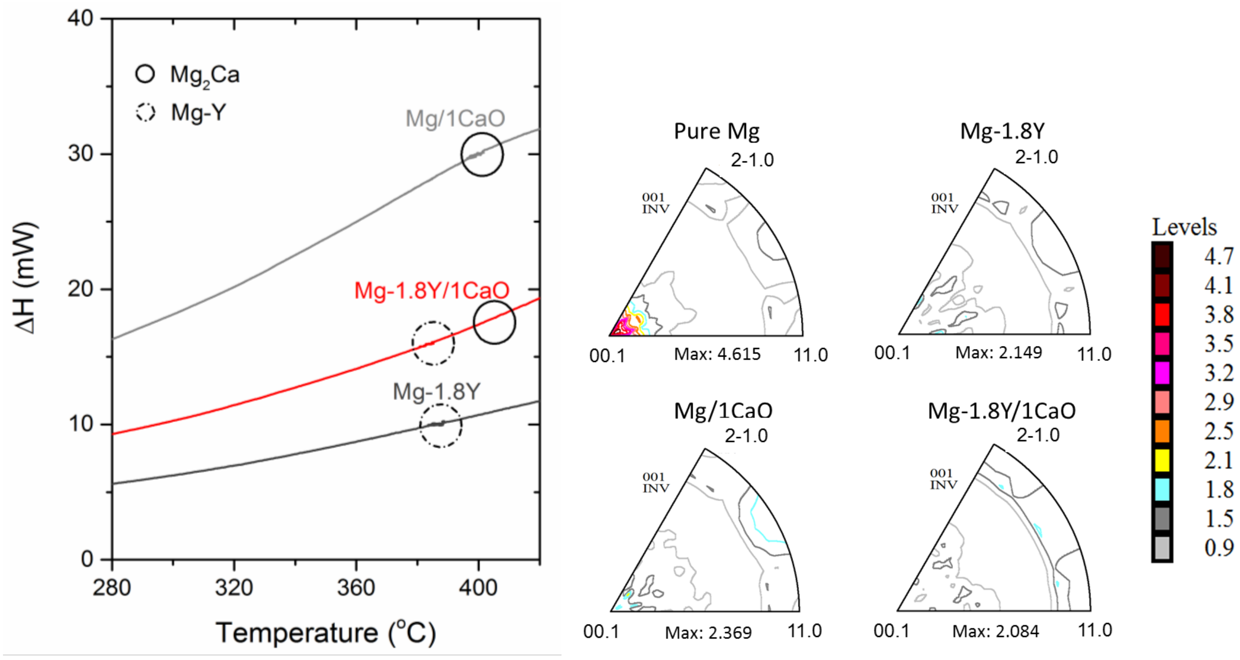

(A) **(B)**

Fig. S3 (A) Differential scanning calorimetric results of Mg-1,8Y, Mg/1CaO and Mg-1.8Y/1CaO nanocomposite. From the curves, it is seen that the reaction occurring at 407°C is prominent only in Mg/1CaO indicative of Mg_2Ca phase while the curve of Mg-1.8Y/1CaO indicates the formation of $Mg_{24}Y_5$ phase; **(B)** Global textures, measured by X-ray diffraction, represented as inverse pole figures for all the materials with maximum intensities given for each. The projected direction is the radial direction (RD).

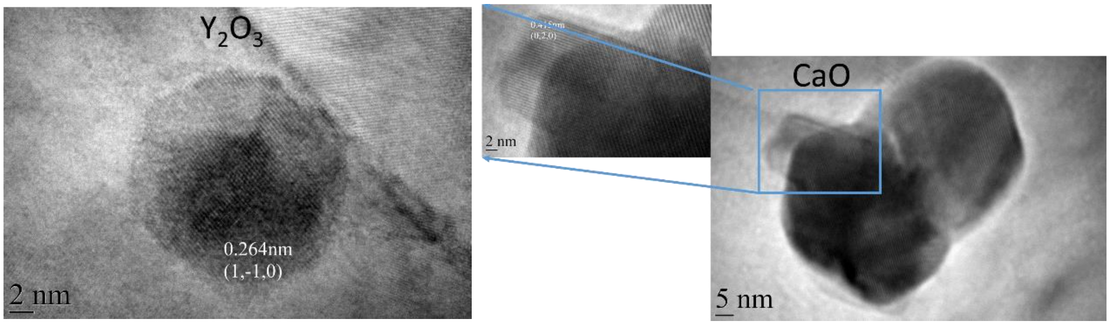

(A) **(B)**

Fig. S4. Transmission electron micrographs of Mg-1.8Y/1CaO samples with energy dispersive spectroscopic analysis on the two different forms of CaO **(A)** indicates the in-situ formed spherical Y_2O_3 nanoparticle**; (B)** the untransformed stable CaO nanoparticle in the matrix.

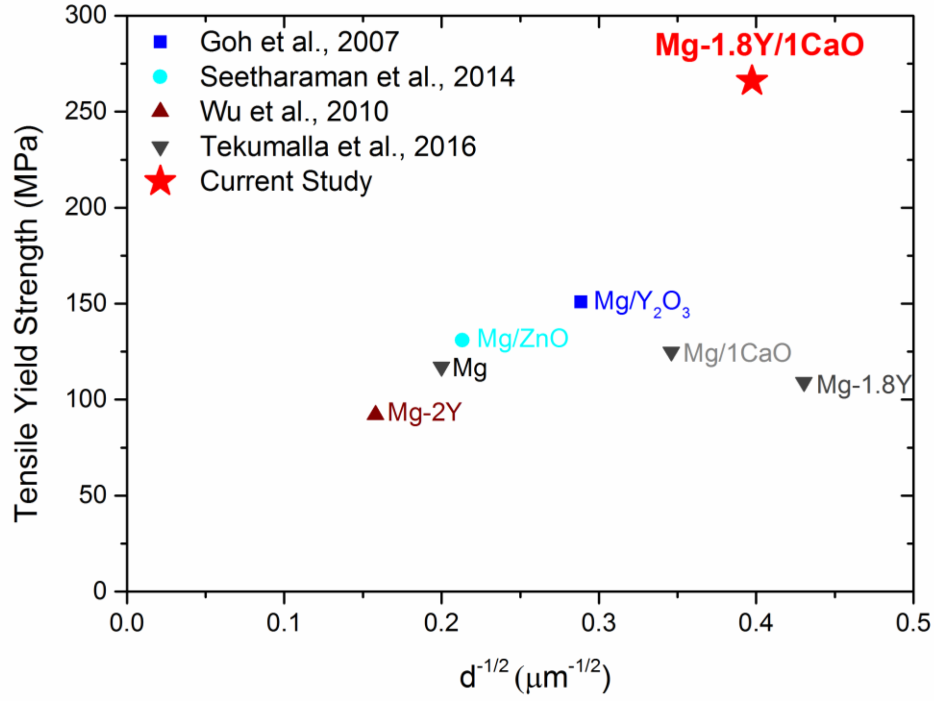

Fig. S5. The plots of tensile yield strength versus inverse square root of the grain diameter indicating the Hall Petch strengthening in Mg-1.8Y/1CaO nanocomposite.

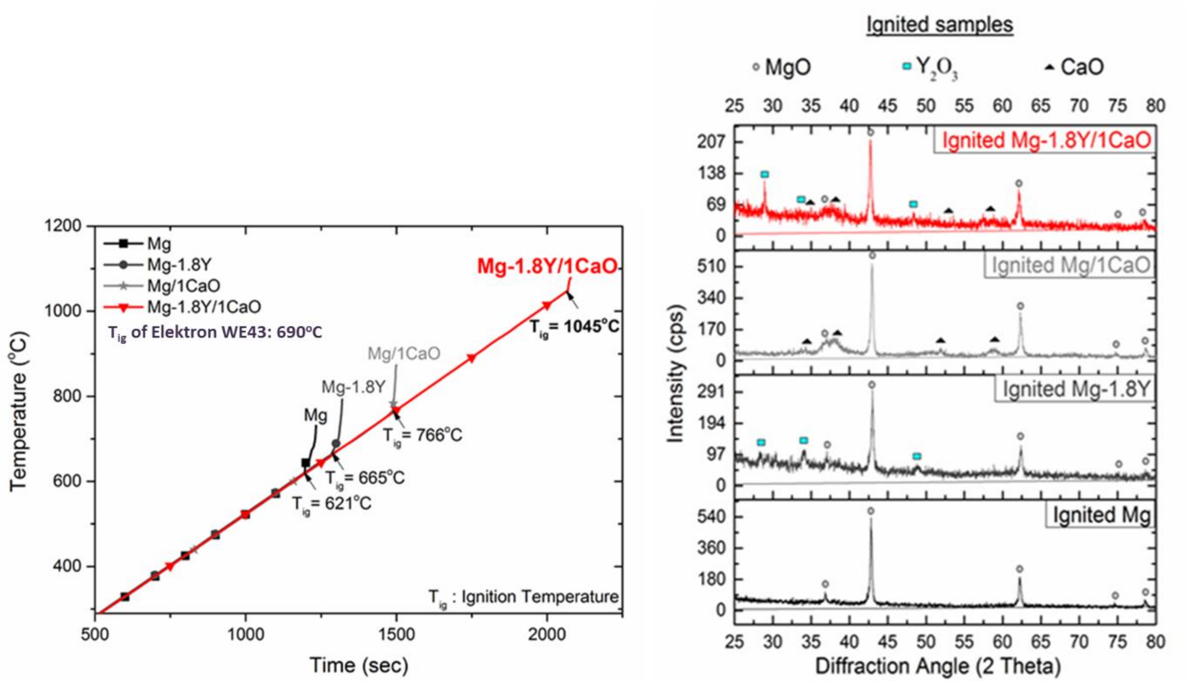

(A) **(B)**

Fig. S6. (A) ; Temperature vs. time plot of the samples tested in TGA at a heating rate of 30°C/min and a sample size of 2×2×1 mm^{3} ; **(B)** XRD of the powder formed after the ignition of samples.

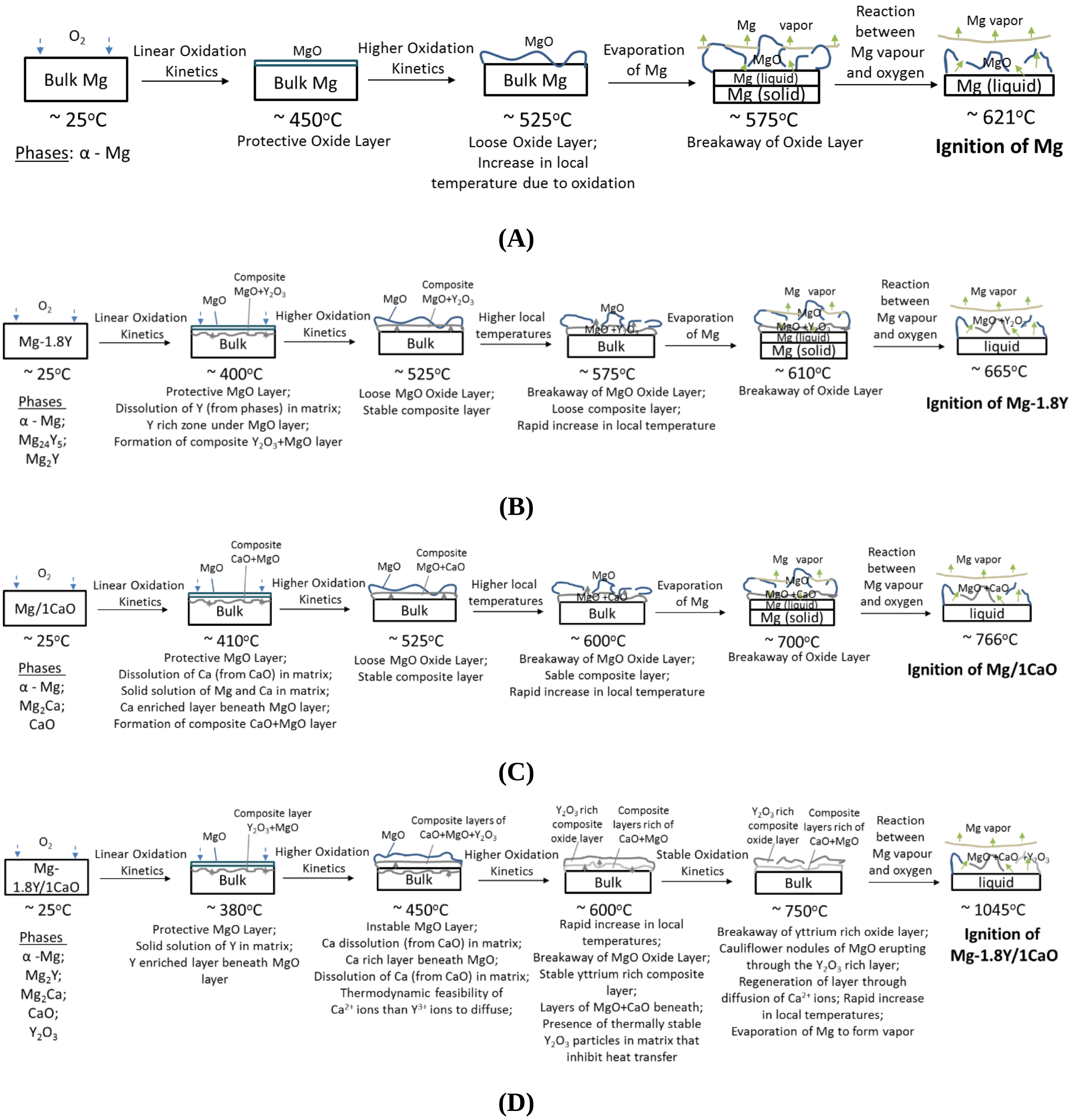

Fig. S7. Detailed ignition mechanisms involved in **(A)** Pure Mg; **(B)** Mg-1.8Y alloy; **(C)** Mg/1CaO nanocomposite and **(D)** Mg-1.8Y/1CaO nanocomposite.

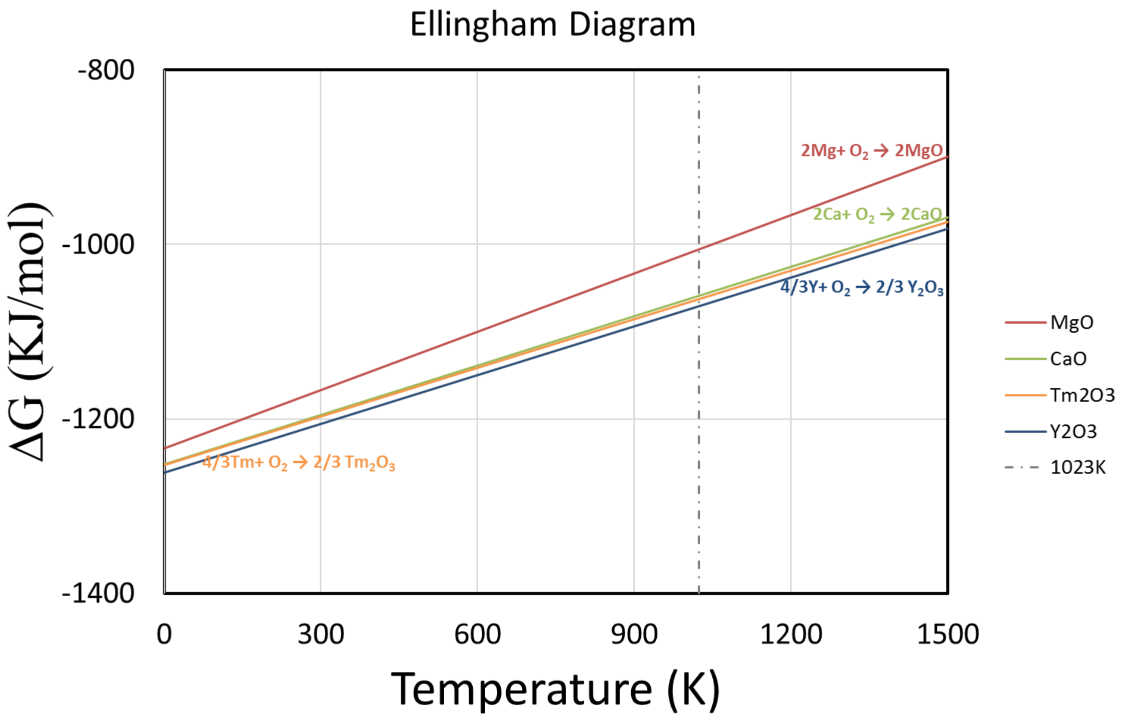

Fig. S8 Ellingham diagram with the Y→Y_2O_3 line and Ca→ CaO line with a marker at 750°C (1023K). Additional lines like Tm→Tm_2O_3 are also shown for thermodynamic comparison with that of Y.

Table S1.
Mechanical Properties and ignition temperatures of all the existing Mg-base [5, 6, 7, 8], Al-base [9, 10], Ti-base [11, 12] commercial alloys and steels [10] with potential application in aerospace and defense sector [13] along with the results of the current study.

	Alloys	Density (g/cc)	TYS (MPa)	Specific Strength	Ductility	Ignition Temperature (°C)
**Current study**	Mg	1.727	117 ± 6	67.75	14 ± 1	621
	Mg-1.8Y	1.734	109 ± 6	62.86	27 ± 2	665
	Mg/1CaO	1.716	119 ± 9	69.35	12 ± 3.5	766
	**Mg-1.8Y/1CaO**	**1.759**	**266 ± 5**	**151.22**	**32 ± 2**	**1045**
**Commercial Mg Alloys**	Pure Mg	1.732	130	75.06	12	615
	AZ31	1.754	175	99.77	15	628
	AZ61	1.777	218	122.68	14	559
	AZ63	1.83	200	109.29	-	573
	AZ91	1.81	230	127.07	12.6	600
	AM50	1.77	230	129.94	10	585
	AM60	1.8	130	72.22	8	525
	ZK40A	1.78	255	143.26	4	500
	ZK51A	1.8	275	152.77	-	552
	ZK60A	1.83	275	150.27	11	499
	Elektron WE43	1.8	250	138.89	14	690
	WE54	1.85	210	113.5	4.5	-
**Commercial Al alloys**	AA2024 - T3	2.78	345	124.10	18	2072
	AA6061 - T6	2.7	255	94.44	8	1877
	AA7075 - T6	2.795	430	153.85	11	2072
**Commercial Ti alloys**	Ti alloy	4.5	-	-	-	1204
	VT5	4.4	863	196.14	10	873
	Ti-6Al-4V (Ti-6-4)	4.429	896	202.30	14	1600
**Commercial Steels**	302 Stainless steel	7.9	275	34.81	50	1399
	1018 Carbon steel	7.87	370	47.01	15	1389
	Carbon steel	7.85	-	-	-	816
	A36 structural ASTM steel	7.8	250	32.05	23	-

Supplementary Reference List

1. Tekumalla S, Shabadi R, Yang C, Seetharaman S, Gupta M. Strengthening due to the in-situ evolution of ß1′ Mg-Zn rich phase in a ZnO nanoparticles introduced Mg-Y alloy. *Scripta Materialia* 2017, **133:** 29-32.

2. Wiese B, Mendis CL, Tolnai D, Stark A, Schell N, Reichel HP, *et al.* CaO dissolution during melting and solidification of a Mg–10 wt.% CaO alloy detected with in situ synchrotron radiation diffraction. *Journal of Alloys and Compounds* 2015, **618:** 64-66.

3. Cullity BD, Weymouth JW. Elements of X-ray Diffraction. *American Journal of Physics* 1957, **25**(6)**:** 394-395.

4. Tekumalla S, Gupta M. An insight into ignition factors and mechanisms of magnesium based materials: A review. *Materials & Design* 2017, **113:** 84-98.

5. Czerwinski F. *Magnesium Injection Molding*. Springer US, 2007.

6. Bettles C, Barnett M. *Advances in Wrought Magnesium Alloys: Fundamentals of Processing, Properties and Applications*. Elsevier Science, 2012.

7. Jayalakshmi S, Gupta M. *Metallic Amorphous Alloy Reinforcements in Light Metal Matrices*. Springer International Publishing, 2015.

8. Lyon BGP. Magnesium Alloys in Aerospace Applications, Past concertns, Current solutions. *Triennial International Aircraft Fire & Cabin Safety Research Conference*; 2007.

9. Kaufman JG. *Properties of Aluminum Alloys: Tensile, Creep, and Fatigue Data at High and Low Temperatures*. ASM International, 1999.

10. Nguyen K, Branch MC. Ignition Temperature of Bulk 6061 Aluminum, 302 Stainless Steel and 1018 Carbon Steel in Oxygen. *Combustion Science and Technology* 1987, **53**(4-6)**:** 277-288.

11. Titanium Industries I. MATERIAL SAFETY DATA SHEET FOR TITANIUM METAL. 2011.

12. Thomas R. Strobridge JCM, Alan F. Clark. Titanium combustion in turbine engines. Colorado: Thermophysical Properties Division, National Enineering Laboratory, National Bureau of Standards, Boulder; 1979.

13. Williams JC, Starke Jr EA. Progress in structural materials for aerospace systems1. *Acta Materialia* 2003, **51**(19)**:** 5775-5799.
